# Role of hyperbaric oxygen therapy in PDGF-BB-mediated astrogliosis in traumatic brain injury rats associated with ERK1/2 signaling pathway inhibition

**DOI:** 10.1186/s40001-023-01062-1

**Published:** 2023-02-25

**Authors:** Guanghui Xiu, Xiuling Li, Qiang Li, Yunyu Yin, Qiqi Tang, Jintao Li, Jiaying Ling, Bin Ling, Ying Yang

**Affiliations:** 1 Affiliated Hospital of Yunnan University, School of Medicine, Yunnan University, Kunming, 650021 Yunnan China; 2 Department of Obstetrics, The First People’s Hospital of Yunnan Province, Kunming, 650100 Yunnan China; 3 Department of Emergency Medicine, Fushun People’s Hospital, Zigong, 643200 Sichuan China; 4 Department of Intensive Care Unit, Affiliated Hospital of North Sichuan Medical College, Nanchong, 637002 Sichuan China; 5 Institute of Neuroscience, Kunming Medicine University, Kunming, 650500 Yunnan China; 6 Kunming Medical University Haiyuan College, Kunming, 650106 Yunnan China; 7 No. 176 Qingnian Road, Wuhua District, Kunming, 650021 Yunnan China

**Keywords:** Hyperbaric oxygen therapy, Traumatic brain injury, Platelet-derived growth factor, Extracellular signal-regulated kinase 1/2, Astrogliosis, Neural regeneration, Glial scar formation

## Abstract

**Background:**

Hyperbaric oxygen (HBO) plays positive roles in the therapy of traumatic brain injury (TBI); however, the mechanism underlying its effects on TBI is largely unknown. The study aims to elucidate the molecular mechanism implicated with the interaction between platelet-derived growth factor-BB (PDGF-BB) and extracellular signal-regulated kinase 1/2 (ERK1/2) signaling pathway, which may play critical roles during HBO treatment both in the astrocyte scratching model in vitro and rat TBI model in vivo.

**Methods:**

Changes in neurological function and wound healing were evaluated using the neurological severity scores (NSS) scale, immunohistochemistry, western blotting, and qRT-PCR, respectively.

**Results:**

The results showed that PDGF-BBi (PDGB interfered with small RNA) dramatically improves neuronal viability in vitro when transfected into the scratched astrocytes derived from the cerebral cortex of neonatal rats. Moreover, in vivo experiments revealed that HBO therapy substantially elevated the NSS scores and simultaneously reduced the mortality in TBI rats, as indicated by the NSS scales. Notably, HBO therapy was found to possess the ability to inhibit glial cell proliferation, promote the regeneration of neurons and synapses, and ultimately facilitate the wound healing, as revealed by immunohistochemistry and glial scar formation found in TBI rats. Importantly, HBO markedly decreased the expression levels of PDGF-BB and ERK1/2. It can clearly be seen that downregulated PDGF-BB and ERK1/2 levels were corresponding with the status of significant amelioration of the therapeutic effect of HBO. Conversely, the upregulation of PDGF-BB and ERK1/2 levels was in line with the opposite effect.

**Conclusion:**

It has been concluded that HBO therapy may play its active role in TBI treatment dependent on astrogliosis inhibition, which may be achieved by downregulating the ERK1/2 signaling pathway mediated by PDGF-BB.

## Background

Traumatic brain injury (TBI), as one of the leading cause of death and disability in young people, has well known that two distinct phases—primary and secondary injury—with a worldwide mortality rate of 20–30% [1–3]. Currently, the major TBI therapeutic strategies mainly comprise neurorehabilitation training and administration of neurotrophic or neuroactive medications. However, there are rigid challenges for beneficial therapeutic effects. For example, for some TBI patients, the loss of motor functions commonly could not recover to the ideal level due to the glial scars produced at the injured site [4, 5]. Furthermore, some other important functions, particularly behavioral, cognitive, and executive functions, could neither be easily recovered after TBI.

It has been demonstrated that PDGF-BB, a subtype of platelet-derived growth factor (PDGF), plays a crucial role in promoting the division and proliferation of connective tissues [6]. Recent studies have revealed that the activation of extracellular signal-regulated kinase l/2 (ERK l/2) is involved in the phosphorylation of some protein molecules that exist in its down-stream PDGF-BB pathway, accompanied by various physiological processes such as the regulation of cellular proliferation, differentiation, and apoptosis [7]. However, there are few reports involving the interaction between ERK1/2 signaling pathway and PDGF-BB following TBI, especially under the treatment by hyperbaric oxygen (HBO). Since the glial scars are one of the major factors influencing the recovery of motor function following TBI, the molecular mechanism under interaction between PDGF-BB and ERK l/2 signaling pathway related to the astrogliosis formation has attracts more and more attention.

HBO therapy had been widely applied in the clinic since the 1960s, which could reduce brain edema and inflammation. In the meantime, it has been revealed that HBO therapy showed promising effects in ameliorating neurological deficits after TBI [8, 9]. In recent years, accumulating evidences indicated that HBO therapy not only promotes the repair of wounds, but also inhibits the excessive proliferation of glial cells, attributed greatly due to its distinctive character to inhibit scar formation, thereby facilitating the neurological repair on this basis [10, 11]. However, the mechanisms through which HBO therapy exerts its neuroprotective role remain to be elucidated.

Our previous study demonstrated that HBO therapy markedly reduces the neurological severity scores (NSS) and ERK1/2 expression in acute TBI rats [12]. Based on this, in this study, we aimed to explore the effects of PDGF-BB on the proliferation of astrogliosis and glial scar formation, under both PDGF-BB over-expressed and interfered state in TBI rat models, treated by HBO. Moreover, the relationship between ERK1/2, an important PDGF-BB downstream signaling molecule, and PDGF-BB and roles of their interaction in the astrogliosis formation were explored and discussed. This will shed new lights on the intensive elucidation of the molecular mechanisms of HBO underlying its actions in TBI rats and bring about optimal therapy for central nervous system (CNS) diseases using HBO therapy.

## Materials and methods

### Construction of HSV-PDGF-BB or HSV-siPDGF-BB vectors

Non-replication herpes simplex virus (HSV) vectors were prepared by deletion of the immediate early genes ICP4, ICP27, and IP34.5, and by insertion of a cassette consisting of CMV promoter and polyA elements or U6 and H1 promoter into the UL41 locus. Rat PDGF-BB coding sequence or two rat PDGF-BB shRNA sequences (sh1 and sh2) according to PDGF-BB gene were cloned into the UL41 locus (including CMV, PDGF-BB gene and polyA or U6, Sh1, Sh2 and H1) of HSV to generate HSV-PDGF-BB or HSV-PDGF-BBi vectors. The success of HSV-PDGF-BB was confirmed using reverse transcription-polymerase chain reaction (RT-PCR). The constructed recombinants were transfected into Vero cells, respectively, and the PDGF-BB expression level in Vero cells was detected by western blotting.

### Culture of cortical astrocytes

Neonatal Sprague–Dawley (SD) rats underwent carotid bloodletting to death and were soaked in 75% ethanol, and then the cerebral cortex was dissected and taken out under the sterile condition. The meninges and hippocampus were removed under the anatomic microscope, and cerebral cortex was separated and kept in a centrifuge tube with D Hanks fluid. The cerebral cortex tissue solution was blown with a pipette (diameter 0.5 mm) until a single cell suspension formed. Subsequently, centrifugation under 1000 rpm/min was carried out for 5 min, and the supernatant was discarded. The cells were suspended in DMEM/F12 medium containing 10% fetal bovine serum and inoculated in a culture flask pretreated with 0.05 g/L polylysine and incubated at 37 ºC in a 5% CO_2_ incubator for 9 days (fresh medium was changed on day 3 and every two days thereafter). Before sub-culturing was conducted on day 9, 0.25% trypsin was used for digestion. The 3rd generation of cells was identified with GFAP antibody and over 95% of cells were positive for GFAP immunoreactivity.

### Wound-healing assay

In wound-healing experiments, the 3rd generation cortical astrocytes were divided into normal group, injury group, and PDGF-BBi group and inoculated in 35 mm diameter petri dishes until 90% confluence was reached. To maintain the consistency of cell damage extent in each group, the monolayer was scratched vertically with a sterile pipette tip in crisscross line and the width of the scratch was kept consistent in all petri dishes. The cells were washed 3 times with phosphate-buffered solution (PBS) to remove non-adherent cells and then replaced with fresh serum-free medium and incubated in a 5% CO_2_ incubator at 37 °C. Photographs of the scratched wounds were recorded at 24 h (h) and 48 h using an inverted microscope. In the injury group, the cell damage model was established according to the method above mentioned. In the PDGF-BBi group, the damaged astrocytes were transfected with HSV-PDGF-BBi.

### Cell proliferation assay

The effect of PDGF-BB and ERK1/2 on cell proliferation of astrocytes was determined by 3-[4, 5-dimethylthiazol-2-yl]-2,5-Diphenylte-trazolium bromide (MTT) assay. Briefly, the astrocytes of Normal group, Injury group, and PDGF-BBi group were inoculated in the 96-well plates in the medium. After 1, 2, 3, 4, 5, and 6 days (d), cells were incubated with MTT solution (5 mg/ml in the culture medium) at 37 °C for 4 h. Dimethyl sulfoxide (DMSO) was then added to the each well, and the plates were oscillated for 10 min. Finally, the optical density (OD) was measured at 490 nm using a microplate reader.

### Immunocytochemistry

In brief, the astrocytes of Normal group, Injury group, and PDGF-BBi group were inoculated in the 96-well plates and added with 3% H_2_O_2_ for 10 min at room temperature. The cells were then washed with PBS and followed by incubation with the blocking solution, PBS containing 5% bovine albumin (BSA), at 37 °C for 30 min. After aspiring the blocking solution, the cells were incubated with the primary antibodies (anti-PDGF-BB, rabbit; anti-ERK1/2, rabbit) at 37 °C for 1 h. Next, discarding the primary antibodies and washing with PBS containing 0.1% Tween-20 (PBST) for 3 times [5 min each], the cells were incubated with anti-rabbit horseradish peroxidase (HRP) at 37 °C for 30 min. Finally, the cells were then washed with PBS for 3 times [5 min each] and stained with DAB solution until yellow–brown color appeared, and then rinsed 3 times with tap water. Observation of the cells was performed using an inverted microscope.

### Animals and grouping

A total of 96 adult male SD rats (weighing 180-200 g) were provided by the Experimental Animal Center of Kunming Medical University and randomly divided into six groups as follows: sham group (n = 16), TBI group (n = 16), HBO group (n = 16), PDGF-BBi group (n = 16), PDGF-BB group (n = 16), and HSV group (n = 16). All procedures were approved by the Ethics Committee of Kunming Medical University (Approval No. KMMU2016004) and were in accordance with the Animal Experiment Guideline of Kunming Medical University.

### Establishment of TBI rat models

TBI rat models were carried out as described by Feeney [13] with slight modifications. Briefly, rats were anesthetized intraperitoneally with phenobarbital sodium (40 mg/kg) and immobilized in prone position. After routine sterilization, the right parietal bone was exposed and a bone hole was drilled 2.5 mm adjacent to the right of the sagittal suture and 1.5 mm posterior to the coronal suture, and a bone window sized 5 mm × 5 mm (25mm^2^) was created using a bone Rongeur to expose the dura mater. An iron cylinder (40 g) was dropped from a height of 15 cm and struck the exposed cranium, causing a moderate contusive injury of the right parietal lobe. Then, the bone window was closed with bone wax, the wound was sutured and sterilized, and penicillin was administered intramuscularly until 3 days postoperatively to prevent infection. In the sham group, only the bone window was opened, with right parietal lobe intact. In the PDGF-BB group, four coordinate points were selected to avoid accidental vascular injury, and each point was injected with 0.5 μl of HSV-PDGF-BB recombinant with a micro syringe. For the PDGF-BBi and HSV groups, HSV-PDGF-BBi recombinant or HSV empty vector was injected through the same coordinates, respectively. Finally, bone wax was used to seal the bone window, and the wound was sutured and disinfected thereafter. Penicillin was injected intramuscularly until 3 days postoperatively to prevent infection.

### HBO therapy

The rats in the HBO, PDGF-BBi, PDGF-BB, and HSV groups were treated with HBO within 1 h after TBI modeling. The rats were treated with pure oxygen for 60 min after entering the chamber under pressure: first, the pressure was raised to 2.0 ATA (0.1 ATA/min) for 20 min, then maintained at 2.0 ATA for 20 min, and slowly decompressed for 20 min at the end. The temperature of the HBO chamber was maintained between 22 and 25 °C, and carbon dioxide was absorbed by an absorption device. Besides, the sham and TBI group rats were also required to be exposed to the HBO chamber for the same time but without undergoing HBO therapy.

### NSS scale

Rats were examined for a NSS scores at 7 days and 14 days after TBI using a five-tiered grading system as follows: 0, normal neurological function; 1, mild neurological deficit (failure to extend contralateral forepaw when lifting tail); 2, moderate neurological deficit (spin longitudinally); 3, severe neurological deficit (falling to the contralateral side); 4, unable to walk spontaneously; and 5, ischemia-related death. Double-blind method was used to assess each group of rats, and the higher the score, the more severe the neurological deficit.

### Tissue harvest

On 7 d and 14 d after TBI, the rats were anesthetized by intraperitoneal injection of phenobarbital sodium (40 mg/kg) and killed by transcardial perfusion, first with cold PBS for 5 min followed by 4% paraformaldehyde solution for 30 min and finally immersed in 0.1 M PBS containing 20% sucrose overnight until the tissues sank to the bottom of bottle. The sections (20 µm thickness) were cut with a freezing microtome (Leica CM1900, Germany) and prepared for immunohistochemistry (n = 4 for each group) and TdT-mediated dUTP nick-end labeling (TUNEL) staining (n = 4 for each group).

For western blotting (n = 4 for each group) and quantitative reverse transcription-polymerase chain reaction (qRT-PCR) analysis (n = 4 for each group), the rats were killed at 7 d and 14 d after TBI, respectively. Rats were anesthetized by intraperitoneal injection with 4% pentobarbital sodium (40 mg/kg) and killed by transcardial perfusion with normal saline. The brain tissues were harvested and homogenized on ice, and the supernatant of tissues was collected and stored at −80 °C.

### Immunohistochemistry

In brief, the sections were rinsed with 0.01 M PBS for three times for 5 min each and soaked in PBS containing 3% H_2_O_2_ for 30 min at room temperature to block the endogenous peroxidase activity. Then, the sections were immersed in 0.01 M PBS containing 5% goat serum and 0.3% TritonX-100 solution at 37 °C for 30 min followed by incubation with primary antibodies (anti-GFAP (rabbit, 1:4000, Chemicon), anti-NeuN (rabbit, 1:500, Chemicon), anti-synaptophysin (SYP) (rabbit, 1:4000, Chemicon)) at 4 °C overnight. After three washes with PBST for 5 min each, the sections were incubated with Reagents I and II (PV-9000 Reagent Kit, Anti-Rabbit/Mouse Poly-HRP IHC Detection Kit, Chemicon, USA) for 30 min each at 37 °C, respectively. Finally, the sections were stained with DAB solution until yellow–brown color appeared and then rinsed three times with tap water. The negative control was performed with 2% goat serum instead of primary antibody. Immunoreactive products were observed and photographed using a light microscope (Leica. DMIRB, Germany).

### TUNEL staining

An in situ cell death detection kit, POD (11684817910, Roche, Germany) was used for the TUNEL staining, and the kit protocol was applied following the dewaxing and dehydration. Briefly, dewaxed tissue sections were predigested with proteinase K for 20 min and incubated in PBS containing 3% H_2_O_2_ for 10 min to block the endogenous peroxidase activity. After two washes with PBS for 5 min each, the sections were incubated with the TUNEL reaction mixture, fluorescein-dUTP (in situ cell death detection kit, POD, Roche, Germany), for 60 min at 37 °C. The sections were then rinsed three times with PBS for 5 min each and incubated with secondary antifluorescein-POD-conjugate for 30 min. After three washes with PBS for 5 min each, 3, 3′-diaminobenzidine tetrahydrochloride (DAB; 11,718,096,001, Roche, Germany) –H_2_O_2_ chromogen was added on the sections and was counterstained with hematoxylin. After rinsing and mounting, the stained images were observed and captured using an inverted light microscope.

### Western blotting

In brief, the protein concentration extracted from the brain tissue samples was measured using a BCA protein assay kit, and the samples were diluted to the same concentration. The 4 × protein extracts and loading buffer (3:1 in volume) were incubated at 100 °C for 5–10 min followed by 10% SDS-PAGE gel electrophoresis and then the membrane was transferred for 2 h. Subsequently, membranes were incubated with primary antibodies anti-PDGF-BB or anti-phosphorylated-ERK1/2 (p-ERK1/2) for 12 h at 4 °C on a shaker. After washing and incubation with secondary antibodies horseradish peroxidase (HRP)-conjugated anti-mouse IgG for 1 h at 4 °C on a shaker, protein bands were displayed using the ECL luminescent kit. The densitometric analysis of the bands is performed using ImageJ software (National Institutes of Health, USA).

### RNA isolation and qRT-PCR

The total RNA was isolated from the injured brain tissue samples using the TRIzol reagent. The lysate of the samples was transferred into an RNase-free EP tube and mixed with chloroform solution, and the tube was placed on ice for 10 min. After centrifugation at 12,000 rpm/min at 4 °C for 15 min, the supernatant was removed and the precipitate transferred to a 1.5 mL RNase-free EP tube. The same volume of isopropanol was added, mixed, and placed on ice for 10 min, followed by centrifugation at 12,000 rpm/min for 3 min at 4 °C, and the supernatant was discarded. After two washes with 75% ethanol, centrifugation was conducted at 12,000 rpm/min at 4 °C for 3 min and the supernatant was discarded. The cellular deposition was reserved. For RNA drying, a total of 45 μL diethyl pyrocarbonate (DEPC) treated water was added, and the tube was placed at room temperature for 3 min. Then, the centrifugation was carried out for 3 min, and the precipitate was then collected. QRT-PCR analysis was subsequently performed using the StepOnePlus (Applied Biosystems), and the reaction system included: 7.5 μL 2× SG Green qPCR Mix + 0.5 μL upstream primer (10 μM) + 0.5 μL downstream primer (10 μM) + 1 μL cDNA + 6 μL DEPC water. The reaction condition was set as follows: predenaturation (95 °C, 10 min), denaturation (95 °C, 20 s), annealing (60 °C, 30 s), extension (60 °C, 30 s), 40 cycles in total. The β-actin was used as an internal control, and the data were analyzed using the comparative Ct method.

### Statistical analysis

The data were entered into SPSS 24.0 software for statistical analysis. The difference between multiple groups was analyzed by one-way ANOVA with Student–Newman–Keuls (SNK) test, and the difference between two groups was analyzed by *t*-test. The results were expressed as the mean ± standard deviation. ^*^P < 0.05 was considered as statistically significant.

## Results

### Validation of vector construction

**Fig. 1:**
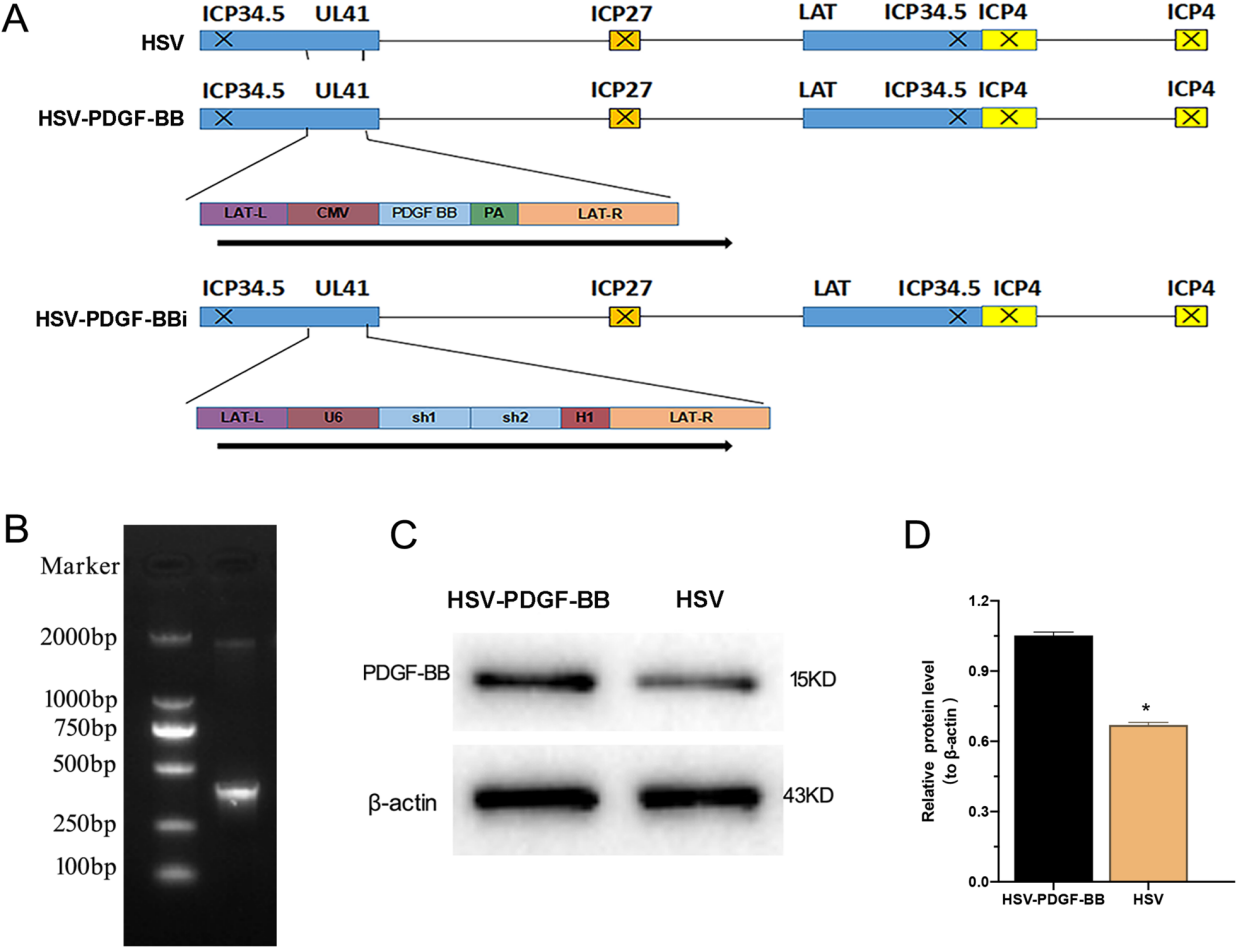
Validation of vector construction. **A** Structures of HSV-PDGF-BB, HSV-PDGF-BBi, and control HSV empty vector. **B** Agarose gel electrophoresis of recombinant plasmids. **C**, **D** Quantitative analysis of western blotting. ^*^P < 0.05, vs. HSV-PDGF-BB

Structures of HSV-PDGF-BB, HSV-PDGF-BBi, and control HSV empty vector are shown in Fig. 1A. The constructed HSV-PDGF-BB recombinant was identified by enzyme digestion, and the bands of about 500 bp were revealed using agarose gel electrophoresis (Fig. 1B). Western blotting showed that the expression level of PDGF-BB protein in Vero cells transfected with HSV-PDGF-BB was higher than that of control HSV empty vector (*P* < 0.05, Fig. 1C, D).

### PDGF-BB and ERK1/2 promoted proliferation of the injured astrocytes in vitro

**Fig. 2:**
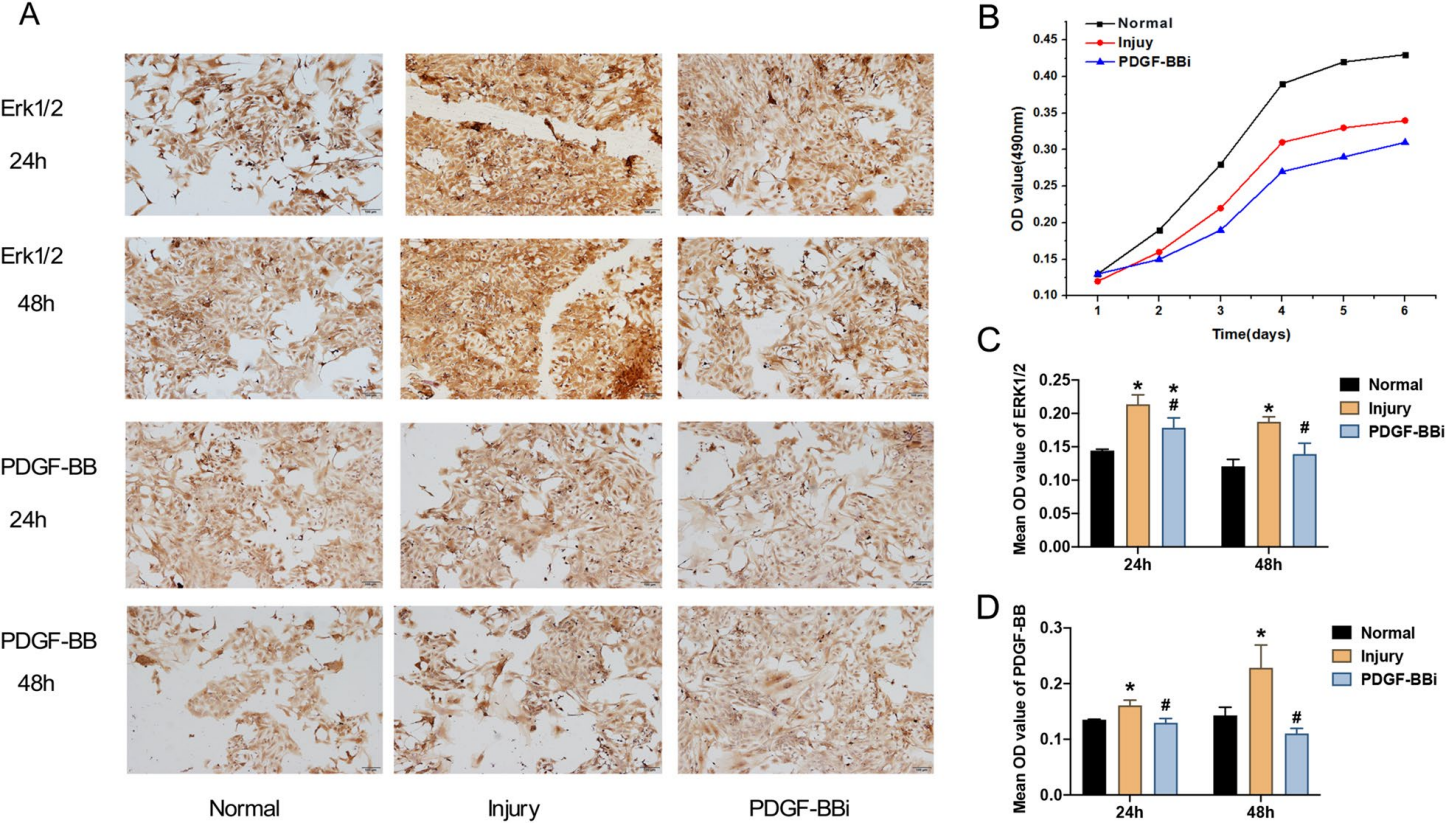
PDGF-BB and ERK1/2 promoted the proliferation of injured astrocytes in vitro. **A** Immunohistochemical staining of PDGF-BB and ERK1/2 at 24 h and 48 h after wound-healing assay (100× , Scale bars = 100 µm). **B** Proliferative ability of injured astrocytes tested by MTT assay. **C**, **D** Comparison of the mean OD values of PDGF-BB and ERK1/2 in the Normal, Injury, and PDGF-BBi groups. ^*^P < 0.05, *vs*. Normal group; ^#^*P* < 0.05, vs. Injury group

**Fig. 3:**
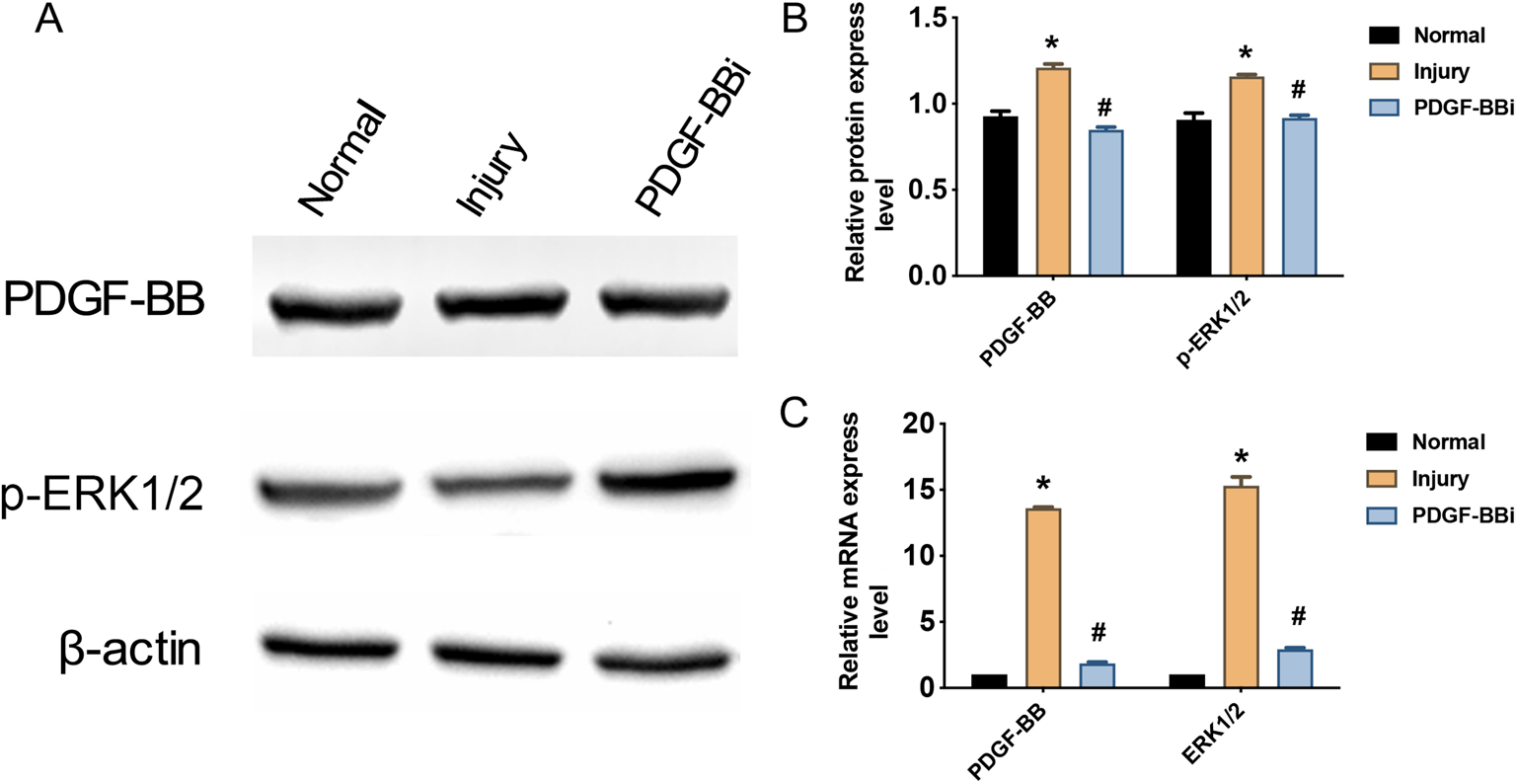
The mRNA and protein expression of PDGF-BB and ERK1/2 in vitro. **A** Western blotting results of PDGF-BB and phosphorylated ERK1/2 (p-ERK1/2) in the Normal, Injury, and PDGF-BBi groups. **B** Comparison of the relative protein expression levels of PDGF-BB and p-ERK1/2 in the Normal, Injury, and PDGF-BBi groups. **C** Comparison of the relative mRNA expression levels of PDGF-BB and ERK1/2 in the Normal, Injury, and PDGF-BBi groups. ^*^P < 0.05, *vs*. Normal group; ^#^*P* < 0.05, vs. Injury group

MTT assay showed that the proliferation ability of the injured astrocytes was substantially weaker than that of the normal group; however, astrocytes in the injured group had markedly greater proliferation ability than that of astrocytes in PDGF-BBi group (Fig. 2A, B). Immunocytochemistry revealed that the mean OD value of PDGF-BB and ERK1/2 significantly increased in the injured group compared with the normal group at 24 h and 48 h after wound-healing assay (*P* < 0.05, Fig. 2C, D). Compared with the injured group, the mean OD value of PDGF-BB and ERK1/2 in the astrocytes of PDGF-BBi group markedly decreased, respectively, at 24 h and 48 h after wound-healing assay (*P* < 0.05, Fig. 2C, )D). Besides, the expression levels of PDGF-BB and phosphorylated ERK1/2 (p-ERK1/2) were determined at 24 h after wound-healing assay (Fig. 3A). Compared with normal group, the protein and mRNA expression levels of PDGF-BB and ERK1/2 (p-ERK1/2 in protein level) were markedly increased in the injured group (*P* < 0.05, Fig. 3B, C). However, the protein and mRNA expression levels of those were significantly decreased in the PDGF-BBi group than those of injury group (*P* < 0.05, Fig. 3B, C).

### HBO therapy reduced NSS and traumatic area, and improved survival rate in TBI rats

**Fig. 4:**
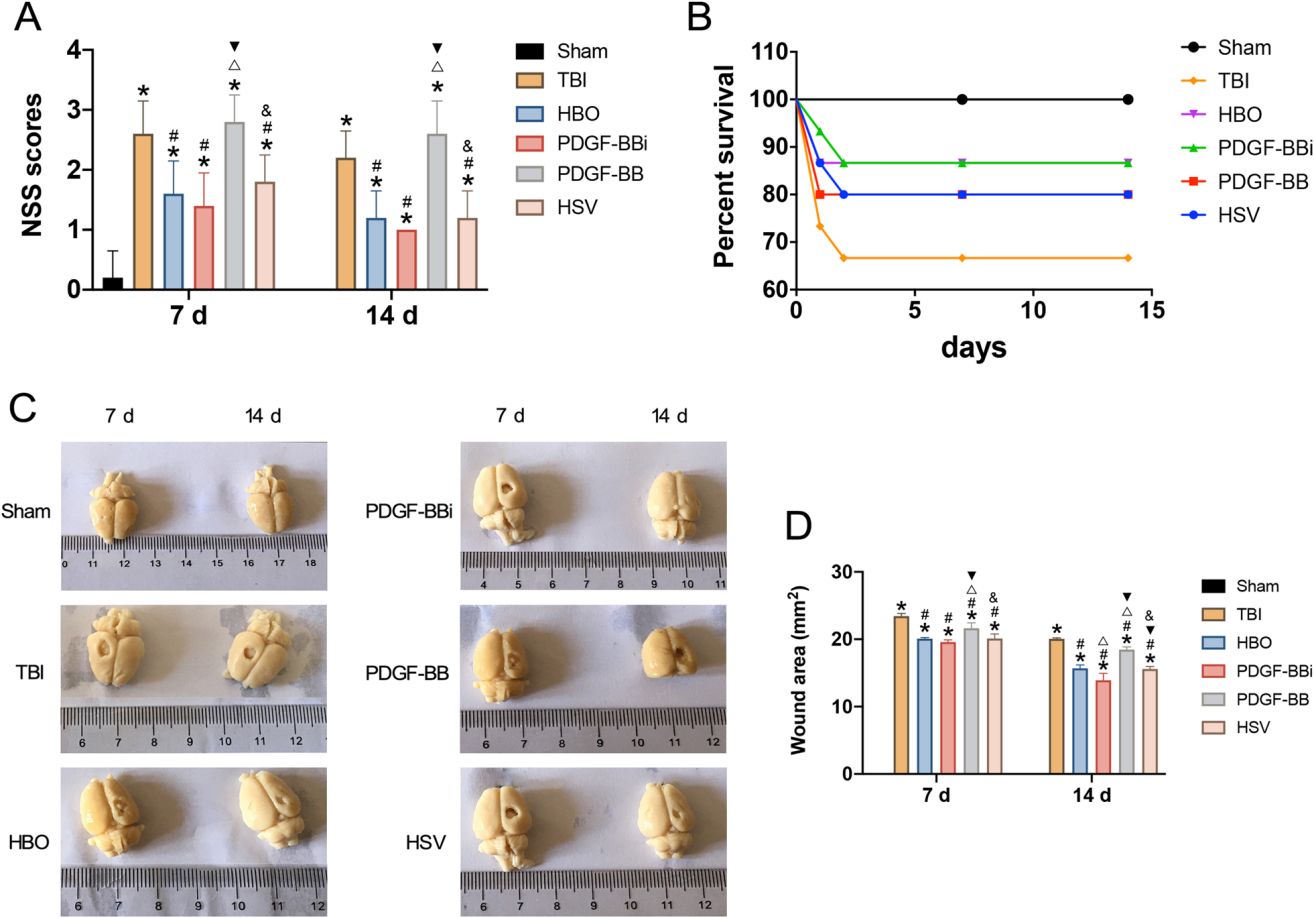
NSS scores, survival rate and traumatic area in the cerebral cortex at 7 days and 14 days after TBI. **A** Comparison of the NSS scores among the six groups. **B** Comparison of the survival rates of six groups. **C**, **D** Measurement and comparison of the wound area in the TBI rats of each group. ^*^P < 0.05, *vs*. Sham group; ^#^*P* < 0.05, *vs*. TBI group; ^△^*P* < 0.05, *vs*. HBO group; ^▼^*P* < 0.05, *vs*. PDGF-BBi group; ^&^*P* < 0.05, *vs*. PDGF-BB group

The NSS scores in the HBO PDGF-BBi and HSV groups were significantly lower than that of TBI group at 7 d and 14 d after TBI modeling, and the survival rate was increased (*P* < 0.05). Compared with HBO group, the NSS scores were significantly increased in PDGF-BB group at 7 d and 14 d after TBI, and the survival rate was also decreased (*P* < 0.05). However, there were no significant differences observed in the NSS scores and survival rate between the PDGF-BBi and HBO groups (*P* > 0.05) (Fig. 4A, B).

The wound area of rats in each group was measured at 7 d and 14 d after TBI. As shown, the traumatic area of the HBO, PDGF-BBi, PDGF-BB, and HSV groups were significantly decreased at 7 d and 14 d after TBI in comparison with that of the TBI group (*P* < 0.05). Compared with the HBO group, PDGF-BBi group had substantially smaller traumatic area, while PDGF-BB group had larger traumatic area (*P* < 0.05). Compared with PDGF-BBi group, PDGF-BB group had markedly larger traumatic area (*P* < 0.05). Besides, the traumatic area in all groups was substantially reduced at 14 d after TBI than that at 7 d after TBI (*P* < 0.05). Compared with the HBO group, the traumatic area of rats in the PDGF-BBi group significantly decreased, while the PDGF-BB group had larger traumatic area (*P* < 0.05). Moreover, in comparison with the PDGF-BBi group, rats in the PDGF-BB group had significantly larger traumatic area (*P* < 0.05) (Fig. 4C, D).

### HBO inhibited the proliferation of astrocytes in TBI rats and promoted the regeneration of neurons

**Fig. 5:**
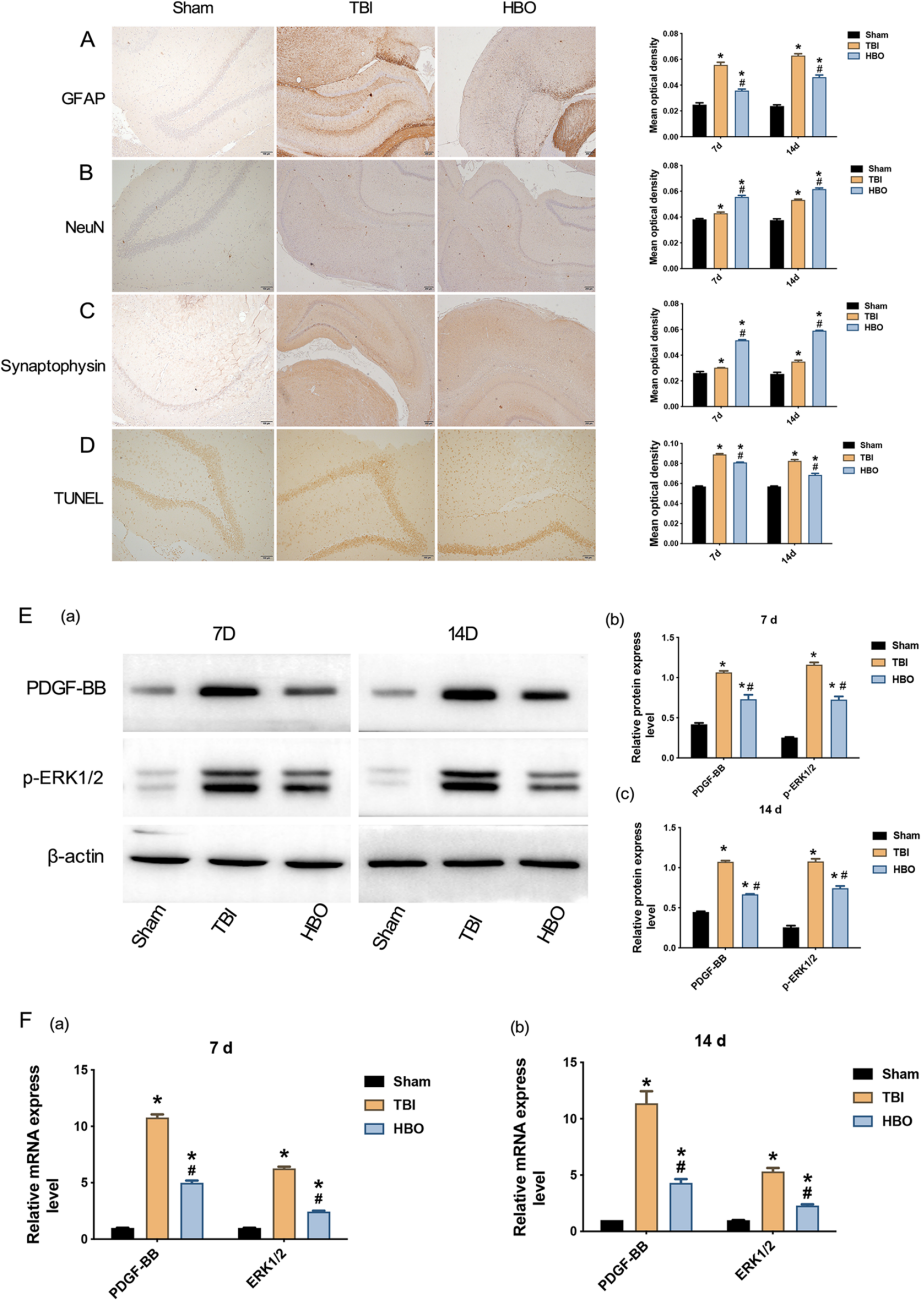
HBO inhibited the proliferation of astrocytes in TBI rats and promoted the regeneration of neurons. **A**–**D** Results of GFAP, NeuN, SYP, and TUNEL staining in the damaged cerebral cortex among the Sham, TBI, and HBO groups (100× , Scale bars = 100um). **E** The western blotting results and the relative protein expression levels of PDGF-BB and phosphorylated ERK1/2 (p-ERK1/2) at 7 d and 14 d after TBI among the Sham, Injury, and HBO groups. **F** Comparison of the relative mRNA expression levels of PDGF-BB and ERK1/2 at 7 d and 14 d after TBI among the Sham, Injury, and HBO groups. ^*^P < 0.05, *vs*. Sham group, ^#^*P* < 0.05, *vs*. TBI group

Immunohistochemistry and TUNEL staining indicated that compared with sham group, the mean OD values of GFAP, NeuN, SYP, and TUNEL staining at 7 d and 14 d after TBI modeling were markedly increased in the TBI group and HBO group (*P* < 0.05, Fig. 5A–D). Compared with the TBI group, the mean OD values of GFAP and TUNEL staining were substantially decreased in the HBO group, while the mean OD values of NeuN and SYP were markedly increased in the HBO group (*P* < 0.05, Fig. 5A–D). Western blotting indicated that at 7 d and 14 d after TBI, the relative protein expression levels of PDGF-BB and p-ERK1/2 were substantially higher in the TBI and HBO groups than those of sham group, and the relative protein expression level of PDGF-BB and p-ERK1/2 in the HBO group was remarkably decreased (*P* < 0.05, Fig. 5E). QRT-PCR analysis demonstrated that the mRNA expression levels of PDGF-BB and ERK1/2 in the TBI group and HBO group were markedly higher than those of sham group, and the mRNA expression levels of PDGF-BB and ERK1/2 in the HBO group were obviously decreased (*P* < 0.05, Fig. 5F).

### PDGF-BB and ERK1/2 were involved in the proliferation of astrocytes in TBI rats and inhibited the regeneration of neurons

**Fig. 6:**
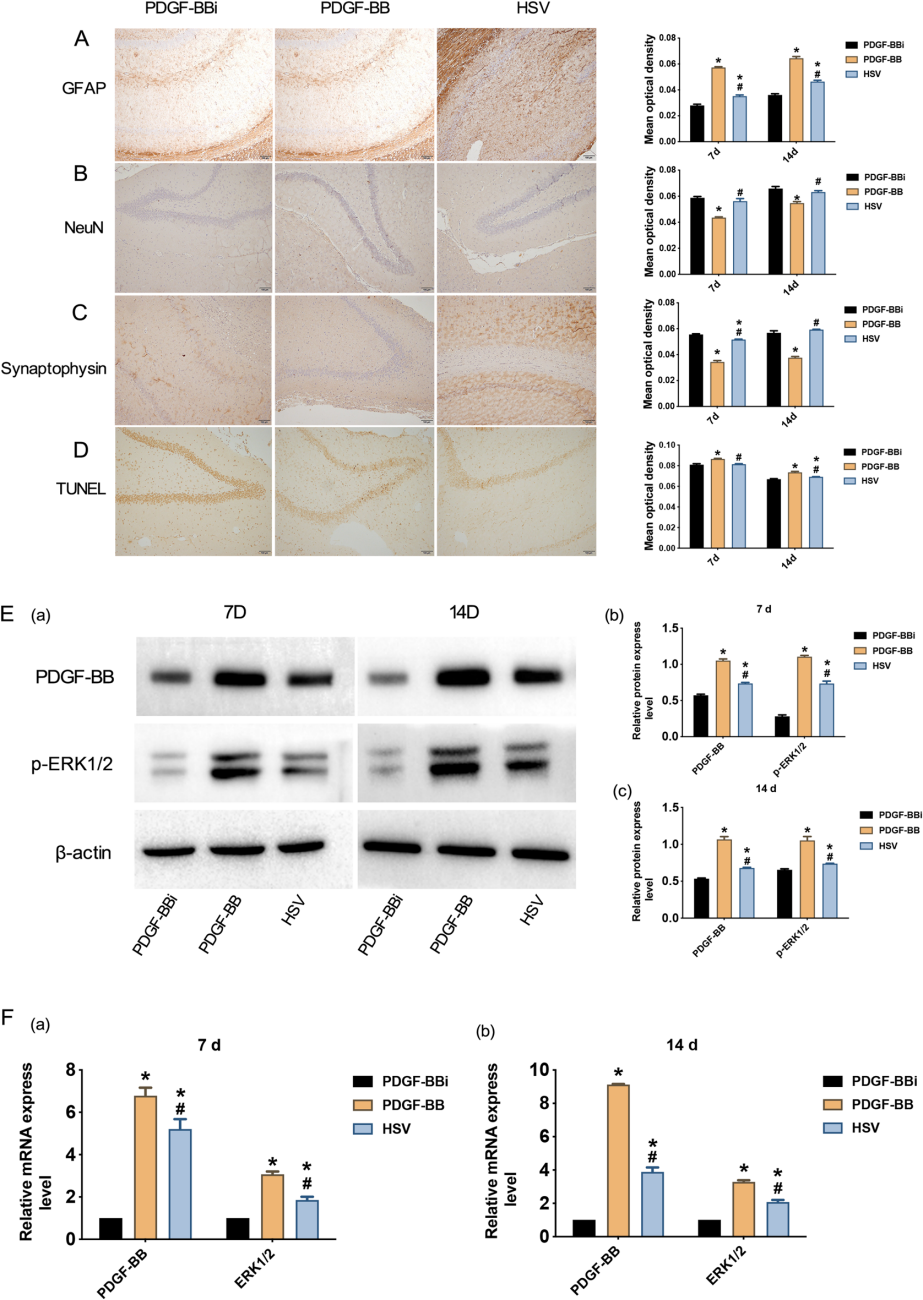
Immunohistochemistry and expression changes of PDGF-BB and ERK1/2 after PDGF-BB regulation. **A**–**D** Results of GFAP, NeuN, SYP, and TUNEL staining in the damaged cerebral cortex among the Sham, TBI, and HBO groups (100 × , Scale bars = 100um). **E** The western blotting results and the relative protein expression levels of PDGF-BB and phosphorylated ERK1/2 (p-ERK1/2) at 7 d and 14 d after TBI among the Sham, Injury, and HBO groups. **F** Comparison of the relative mRNA expression levels of PDGF-BB and ERK1/2 at 7 days and 14 days after TBI among the Sham, Injury, and HBO groups. ^*^P < 0.05, vs. PDGF-BBi group; ^#^*P* < 0.05, vs. PDGF-BB group

As shown, the mean OD values of GFAP and TUNEL staining at 7 d and 14 d after TBI were significantly increased in the PDGF-BB group than those of PDGF-BBi group, while the mean OD values of NeuN and SYP were markedly decreased in the PDGF-BBi group (*P* < 0.05, Fig. 6A–D). Besides, the mean OD values of GFAP at 7 d and 14 d after TBI and the mean OD values of TUNEL staining at 14 d after TBI were significantly higher in the HSV group than those of PDGF-BBi group, while the mean OD values of SYP at 7 d after TBI were markedly lower in the HSV group (*P* < 0.05, Fig. 6A, C, D). However, compared with the PDGF-BBi group, the mean OD values of NeuN at 7 d and 14 d were lower in the HSV group, while the mean OD values of SYP at 14 d and the mean OD values of TUNEL staining at 7 d after TBI were higher in the HSV group (*P* > 0.05, Fig. 6B, C, D). Compared with the PDGF-BB group, the mean OD values of GFAP and TUNEL staining at 7 d and 14 d after TBI were obviously decreased in the HSV group, while the mean OD values of NeuN and SYP were substantially increased (*P* < 0.05, Fig. 6A–D). Western blotting indicated that at 7 d and 14 d after TBI modeling, the relative protein expression levels of PDGF-BB and p-ERK1/2 in the PDGF-BB group and HSV group were substantially higher than those of PDGF-BBi group (*P* < 0.05, Fig. 6E). Moreover, the relative protein expression levels of PDGF-BB and p-ERK1/2 at 7 d and 14 d after TBI in the HSV group were markedly decreased in comparison with the PDGF-BB group (*P* < 0.05, Fig. 6E). Similarly, the qRT-PCR analysis indicated that the relative mRNA expression levels of PDGF-BB and ERK1/2 at 7 d and 14 d after TBI in the PDGF-BB and HSV groups were notably increased than that of PDGF-BBi group; the relative mRNA expression levels of PDGF-BB and ERK1/2 in the HSV group were remarkably decreased in comparison with the PDGF-BB group (*P* < 0.05, Fig. 6F).

## Discussion

TBI is the most common traumatic disease of CNS with a high disability and mortality rate, mainly due to the diffuse necrosis of neurons, nerve protrusion fracture and apoptosis caused by trauma. The main treatment for clinical TBI includes neurological rehabilitation training and neurotrophic or neuroactive drugs. But because of the glial scars in the injured sites, TBI patients inevitably suffer from some forms of permanent disability [4, 14]. It is well known that astrocytes play an indispensable role in CNS trauma and disease. One stage of wound healing after trauma is the formation of fibrous glial scar [15, 16]. Recently, some studies revealed that astrocytes are rapidly activated to form reactive astrocytes after TBI, which could prevent damage progression and promote repair [17].

In this study, we used TBI rat model to reveal the effects of HBO therapy on the PDGF-BB-mediated astrogliosis in the TBI rats associated with ERK1/2 signaling pathway inhibition. Our findings demonstrated that PDGF-BB and ERK1/2 promoted proliferation of the injured astrocytes in vitro, HBO therapy reduced NSS and traumatic area and improved survival rate in the TBI rats. Furthermore, it was found that HBO therapy inhibited the proliferation of astrocytes in TBI rats and promoted the regeneration of neurons, and PDGF-BB and ERK1/2 were involved in the proliferation of astrocytes in the TBI rats and inhibited the regeneration of neurons.

Platelet-derived growth factor-BB (PDGF-BB) is a growth factor beneficial to the glial proliferation, and is upregulated in brain tissues after TBI [18, 19]. Previously, the concrete role of PDGF-BB as a growth factor is ambiguous; it is only known as a kind of nerve growth factor, may be related to the glial proliferation, and plays some certain role in the neuroprotection. Subsequently, PDGF-BB, linked to the ERK1/2 signaling pathway, has synergistic actions with ERK1/2 after brain injury was found. In this study, we found that PDGF-BB and ERK1/2 could be activated to dramatically induce astrocytes to further proliferate. However, PDGF-BB inhibition using SiRNA method could attenuate the proliferation of astrocytes with ERK1/2 reduced expression. In TBI model, rats with PDGF-BB over expression level had significantly larger wound area and higher ERK1/2 expression level when compared with those that with PDGF-BB interference. Therefore, it has been preliminarily concluded that the function of PDGF-BB as a nerve growth factor is most likely involved in the modulation of the astrocytes proliferation accompanied by ERK1/2, the pathway of its downstream, and affects the glial scar formation. Detailly, PDGF-BB interference inhibits to astrogliosis, ultimately contributes to the motor function amelioration after TBI. The most likely molecular mechanism underlying PDGF-BB, combined with ERK1/2, plays its role in functional recovery of TBI, which is the primary finding in this study, elucidating the concrete mechanism of PDGF-BB playing in the TBI rats. Because in the previous studies, the action mechanism of PDGF-BB has not been elucidated clearly, our study sheds a new light on the clarification of the action mechanism of PDGF-BB, which pave a novel way for the PDGF-BB gene intervening strategies for the future therapy of TBI.

HBO is an effective non-traumatic rehabilitation therapy for the treatment of brain injury. It promotes the recovery of neurological function by increasing the oxygen metabolism, thereby increasing the survival rate and reducing the sequelae of brain injury, has been widely accepted in clinical practice. In vitro experiments confirmed that HBO elevated the differentiation rate from neural stem cells (NSCs) into neurons and in the meantime reduced the one from NSCs into astrocytes [20, 21]. As above mentioned, astrocyte proliferation and scar formation are not conducive to the functional recovery of the nervous system. This is confirmed by the present study that HBO therapy could ameliorate the motor function of spinal cord injury rats and reduce the inflammatory response and glial scar formation by inhibiting inflammatory related factors and glial scar related components [22]. In addition, our previous studies found that HBO therapy significantly attenuated the expression of PDGF-BB and ERK1/2 in TBI rats. The present study revealed HBO therapy’s beneficial roles in improvement of neurological function, expressed as the markedly reduction of astroglia scar formation and increase in neuronal regeneration, which is dependent on ERK1/2 pathway inhibition and PDGF-BB downregulation. These were confirmed by PDGF-BB interference using siRNA method. Importantly, HBO therapy played a crucial role in the improvement in different parameters of neuronal functional elevation, including neuronal and glial cell morphology, pathophysiology of the brain and key gene expression after TBI. All the above contributed substantially to the amelioration of neurological function, manifested by an elevation of NSS scores. These changes were accordant with the decreased expression of a PDGF-BB, whose high expression has been widely considered to be tightly related to the occurrence of the astrogliosis. So, it has been concluded that HBO therapy in this study markedly ameliorated the neurological function by astrogliosis inhibition, revealed by in vitro astrocyte proliferative experiments, thereby contributing greatly to the NSS recovery after TBI.

Furthermore, in vitro experiments were also carried out to elucidate the possible molecular mechanisms underlying the beneficial effect of HBO therapy. It was found that PDGF-BB-inhibition resulting in the proliferative number of astrocytes significantly decreased, which was accordant with a status of less glial scar formation. These results suggest that both HBO and gene interventional strategies based on PDGF-BB downregulation could be used in sooner future for the modulation of neuronal damage after TBI or other brain injuries.

Although in our study, it has been revealed that the action mechanism of the HBOT, which visibly ameliorated the locomotive function of TBI rats, was mainly dependent on the inhibition of the PDGF-BB pathway and tightly associated with the synergic changes of its down streaming key molecule-ERK1/2. However, there are still some limitations about this study, which will be paid more attention in future studies to further strengthen the persuasion of our outcomes. First, to intensively elucidate the detailed roles of PDGF-BB in the inhibition of the locomotive functional recovery of the TBI rats, especially in facilitating the formation of the glial scar, PDGF-BB gene knock-out animal model would be used. Under this condition, more convictive results can be obtained. Second, there existed a synergetic change relationship between PDGF-BB and ERK1/2, whereas the concrete interaction and relationship between them are still ambiguous. We only revealed the change tendency between them under HBO treatment. But the specific and clear relationship between them awaits elucidation, including under the treatment of other neuroprotective reagents. Third, the role of astrogliosis in the locomotive functional recovery has been preliminarily interpreted. However, it is not enough to confirm this. More experiments and evidences are still needed to support the notion that how and what astrogliosis affects on the recovery of the function of the motor in TBI rats. So, some intensive works are needed before the molecular mechanism of PDGF-BB mediated by ERK1/2 in TBI rats is confirmed.

## Conclusion

Taken together, revealed by both in vivo and in vitro experiments, the present study has been demonstrated that inhibiting the expression of PDGF-BB and ERK l/2 could suppress the formation of glial scar and thereby improved the functional recovery, especially in the motor functions of rats after TBI.

## Data Availability

The datasets used and/or analyzed during the current study are available from the corresponding author on reasonable request.

## Abbreviations

HBO: Hyperbaric oxygen
TBI: Traumatic brain injury
PDGF-BB: Platelet-derived growth factor-BB
ERK1/2: Extracellular signal-regulated kinase 1/2
NSS: Neurological severity scores
qRT-PCR: Quantitative real-time polymerase chain reaction
CNS: Central nervous system
HSV: Herpes simplex virus
PBS: Phosphate buffered solution
MTT: 3-(4,5-Dimethylthiazol-2-yl)-2,5-Diphenylte-trazolium bromide
DMSO: Dimethyl sulfoxide
OD: Optical density
PBST: PBS containing 0.1% Tween-20
TUNEL: TdT-mediated dUTP nick-end labeling
HRP: Horseradish peroxidase
DAB: 3,3′-Diaminobenzidine tetrahydrochloride
DEPC: Diethyl pyrocarbonate
NSCs: Neural stem cells

## References

[1] Salehi A, Zhang JH, Obenaus A (2017). Response of the cerebral vasculature following traumatic brain injury. J Cereb Blood Flow Metab.

[2] Sivandzade F, Bhalerao A, Cucullo L (2019). Cerebrovascular and neurological disorders: protective role of NRF2. Int J Mol Sci.

[3] Vella MA, Crandall ML, Patel MB (2017). Acute management of traumatic brain injury. Surg Clin North Am.

[4] Dang B, Chen W, He W, Chen G (2017). Rehabilitation treatment and progress of traumatic brain injury dysfunction. Neural Plast.

[5] Moeendarbary E, Weber IP, Sheridan GK, Koser DE, Soleman S, Haenzi B (2017). The soft mechanical signature of glial scars in the central nervous system. Nat Commun.

[6] Meier Bürgisser G, Evrova O, Calcagni M, Scalera C, Giovanoli P, Buschmann J (2020). Impact of PDGF-BB on cellular distribution and extracellular matrix in the healing rabbit Achilles tendon three weeks post-operation. FEBS Open Bio.

[7] Liu X, Wang X, Chen L, Shi Y, Wei Y (2018). Effects of erythromycin on the proliferation and apoptosis of cultured nasal polyp-derived cells and the extracellular signal-regulated kinase (ERK)/mitogen-activated protein kinase (MAPK) signaling pathway. Med Sci Monit.

[8] Jiang Y, Chen Y, Huang C, Xia A, Wang G, Liu S (2021). Hyperbaric oxygen therapy improves neurological function via the p38-MAPK/CCL2 signaling pathway following traumatic brain injury. NeuroReport.

[9] Sahni T, Jain M, Prasad R, Sogani SK, Singh VP (2012). Use of hyperbaric oxygen in traumatic brain injury: retrospective analysis of data of 20 patients treated at a tertiary care centre. Br J Neurosurg.

[10] Xing P, Ma K, Li L, Wang D, Hu G, Long W (2018). The protection effect and mechanism of hyperbaric oxygen therapy in rat brain with traumatic injury. Acta cirurgica brasileira.

[11] da Silva SC, Feres O, da Silva BP, Machado HR, Menezes-Reis R, Araujo JE (2018). Hyperbaric oxygen therapy reduces astrogliosis and helps to recovery brain damage in hydrocephalic young rats. Child's Nerv Syst.

[12] Sun J, Ling B, Li B-Q, Gu Y-F, Gu G-H, Dan Q-Q (2011). Effects of hyperbaric oxygen treatment on expression of ERK1/2 and neurological function in rats with traumatic brain injury. Chin J Traumatol.

[13] Feeney DM, Boyeson MG, Linn RT, Murray HM, Dail WG (1981). Responses to cortical injury: I. Methodology and local effects of contusions in the rat. Brain Res.

[14] Dams-O'Connor K, Ketchum JM, Cuthbert JP, Corrigan JD, Hammond FM, Haarbauer-Krupa J (2020). Functional outcome trajectories following inpatient rehabilitation for TBI in the United States: a NIDILRR TBIMS and CDC interagency collaboration. J Head Trauma Rehabil.

[15] Renault-Mihara F, Mukaino M, Shinozaki M, Kumamaru H, Kawase S, Baudoux M (2017). Regulation of RhoA by STAT3 coordinates glial scar formation. J Cell Biol.

[16] Tran AP, Warren PM, Silver J (2018). The biology of regeneration failure and success after spinal cord injury. Physiol Rev.

[17] Laug D, Huang TW, Huerta NAB, Huang AY, Sardar D, Ortiz-Guzman J (2019). Nuclear factor I-A regulates diverse reactive astrocyte responses after CNS injury. J Clin Investig.

[18] Kempuraj D, Ahmed ME, Selvakumar GP, Thangavel R, Raikwar SP, Zaheer SA (2021). Acute traumatic brain injury-induced neuroinflammatory response and neurovascular disorders in the brain. Neurotox Res.

[19] Kyyriäinen J, Ekolle Ndode-Ekane X, Pitkänen A (2017). Dynamics of PDGFRβ expression in different cell types after brain injury. Glia.

[20] Chen C, Yang Y, Yao Y (2019). HBO promotes the differentiation of neural stem cells via interactions between the Wnt3/β-catenin and BMP2 signaling pathways. Cell Transplant.

[21] Ding Y, Yao P, Hong T, Li H, Zhu Y, Han Z (2018). The analgesic effect of early hyperbaric oxygen treatment in chronic constriction injury rats and its influence on nNOS and iNOS expression and inflammatory factor production. Mol Pain.

[22] Zhou Y, Dong Q, Pan Z, Song Y, Su P, Niu Y (2019). Hyperbaric oxygen improves functional recovery of the injured spinal cord by inhibiting inflammation and glial scar formation. Am J Phys Med Rehabil.

